# Another Unsuccessful Effort

**Published:** 1896-04

**Authors:** 


### ANOTHER UNSUCCESSFUL EFFORT.

Other pages of this issue will inform our readers of the failure to obtain successful army legislation at the hands of the present Congress. With the most thoroughly organized support through the efforts of an untiring and hard-working committee, we are forced to accept the ultimatum that no legislation can be obtained at this time that will grant rank to our members in the army. The committee has decided to allow the bill to rest in the hands of the House Military Committee whose favorable support it had commanded, and to wait until another session, when it may be enabled to obtain a greater unity of action on the part of the army officers. With the support of General Miles and Adjutant-General Ruggles to rely upon, the committee hopes that at the next session the support of the Quartermaster-General may be obtained; and if this is accomplished, the recognition will come to our profession, with all the support and aid that is necessary to insure it the most thorough enforcement in the interests of a long-suffering and patient profession. The members of our calling with one accord will join the JOURNAL in extending to the committee and others a grateful appreciation of their services.

